# Development, characterization and *in vivo* zinc absorption capacity of a novel soy meal hydrolysate-zinc complexes

**DOI:** 10.3389/fnut.2023.1211609

**Published:** 2023-07-06

**Authors:** Rongxin Wang, Meijun Ye, Suyin Zhu, Qingzhu Zeng, Yang Yuan

**Affiliations:** School of Chemistry and Chemical Engineering, Guangzhou University, Guangzhou, China

**Keywords:** soy protein, zinc absorption, hydrolysate, complexes, mRNA expression levels, Zn-related enzymes

## Abstract

**Background:**

Zinc is an essential trace element for the human body. Recently, a novel Zn-binding peptide, Lys-Tyr-Lys-Arg-Gln-Arg-Trp (PP), was purified and identified from soy protein hydrolysates with high Zn-binding capacity (83.21 ± 2.65%) by our previous study. The preparation of soy meal hydrolysates (SMHs)-Zn complexes is convenient and low-cost, while PP (Lys-Tyr-Lys-Arg-Gln-Arg-Trp)-Zn complexes have a higher coordination rate but a relatively high cost. The aim of this study was to investigate the effect of soy meal hydrolysates (SMHs)-Zn complexes on zinc absorption in mice model, and synthetic soy peptide (PP)-Zn complexes with high Zn-binding capacity were used as control. Firstly, SMHs were prepared by enzymolysis, and the PP (Lys-Tyr-Lys-Arg-Gln-Arg-Trp) were synthesized based on previous studies. The binding mechanism of soy hydrolysates and zinc was analyzed by spectral analysis. Furthermore, the cytotoxicity of the SMHs-Zn complexes was also studied using the CCK-8 method. The effect of zinc absorption was evaluated based on Zn content, total protein and albumin content, relevant enzyme system, and the PeT1 and ZnT1 mRNA expression levels.

**Result:**

The result showed that zinc was bound with carboxyl oxygen and amino nitrogen atoms on SMHs, with hydrophobic and electrostatic interactions as auxiliary stabilizing forces. SMHs-Zn were proved to have great solubility and a small particle size at different pH values, and it showed a beneficial effect on Caco-2 cells growth. Moreover, it was proved that SMHs-Zn and PP-Zn could increase the levels of zinc and the activity of Zn-related enzymes in mice. SMHs-Zn possessed higher PepT1 and ZnT1 mRNA expression levels than PP-Zn in the small intestine.

**Conclusion:**

SMHs-Zn with a lower Zn-binding capacity had similar effects on zinc absorption in mice as PP-Zn, suggesting that the bioavailability of peptide-zinc complexes in mice was not completely dependent on their Zn-binding capacity, but may also be related to the amino acid composition.

## 1. Introduction

Zinc ion (Zn), an essential trace element, is well recognized as a key component of cell growth, protein synthesis, and enzymatic and metabolic processes in the human body (1, 2). It also plays an important role in the immune system, protecting the cellular components of organisms from oxidative damage (3). Additionally, zinc deficiency can cause serious health problems, including growth retardation, cognitive impairment, loss of appetite, and immune dysfunction (4). However, traditional Zn supplements (inorganic Zn mineral salts such as ZnSO_4_) are not suitable for long-term intake. Because it is easy to form an insoluble complex with phytic acid and dietary fiber *in vivo* and exhibited low bioavailability (5).

Recent studies found that peptide-Zn complexes were more easily absorbed by the small intestine due to their high Zn solubility under specific pH conditions and had better bioavailability than traditional Zn supplements (6). Food-derived peptides are the most commonly used way to prepare peptide-Zn complexes, which are easily obtained, low-cost, and have significant bioavailability (7). The investigation showed that mung bean peptide, oyster peptide, pollock peptide, and tilapia peptide had been used to complex with Zn, and the binding mechanism and *in vitro* digestion were characterized (8–11). It was found that peptide-Zn complexes could increase the solubility of Zn in simulated gastrointestinal digestion and then improve the bioavailability of Zn *in vitro* (9). To improve the binding ability of Zn, the purified peptides with high Zn-binding capacity were always isolated from food-derived protein hydrolysates (12). Nevertheless, it has not been confirmed whether purified peptide-Zn complexes are more bioavailable than hydrolysate-Zn complexes. Plainly, to narrow this knowledge gap, the investigations about the effect of soy meal hydrolysates (SMHs)-Zn complexes on zinc absorption *in vivo* and synthetic soy peptide (PP)-Zn complexes used as a control *in vivo* are highly needed.

Defatted soy meal is the high-protein solid residue after oil extraction from soy and can be an important resource for obtaining high-quality protein due to its rich nutritional value. The resource of soy meal is extremely rich in China, but it is mainly used in the feed industry with relatively low utilization and economic efficiency because it is partly denatured and could not be absorbed effectively by the human body (13). After soy meal is hydrolyzed, however, it has certain functional properties such as antioxidant activity and anti-exercise-fatigue effect (13, 14). In previous studies, a series of value-added hydrolysate products were developed from soybean meal (15). Researchers had also focused on the improved bioavailability due to the formation of the peptide in soy meal hydrolysates (16, 17). Using soy meal hydrolysates-Zn complexes is a green and efficient method to enhance dietary zinc bioavailability and is worth exploring in depth (5). Recently, a novel Zn-binding peptide, Lys-Tyr-Lys-Arg-Gln-Arg-Trp (KYKRQRW), was purified and identified from soy protein hydrolysates by our previous study (12).

The study aimed to study the effect of soy meal hydrolysates (SMHs)-Zn complexes on zinc absorption in mice model. And synthetic soy peptide (PP)-Zn complexes with high Zn-binding capacity (83.21 ± 2.65%) based on previous studies were synthesized and used as a control (12). Firstly, the Ultraviolet spectrum (UV), Fourier transform infrared spectroscopy (FTIR), Fluorescence spectrophotometer, Dynamic light scattering (DLS), Scanning electron microscope (SEM), and energy dispersion spectrum (EDS) were applied to characterize the formation mechanism and the cytotoxicity of SMHs-Zn complexes were studied using the CCK-8 method (Caco-2 cells). Furthermore, the absorption efficiencies of SMHs-Zn complexes and PP-Zn complexes were compared in mice, and the absorption mechanism of samples in the small intestine and its effects on the growth and related enzyme systems of mice were investigated. Biochemical kits were used to determine the related enzyme activity in mice. The qPCR was employed to measure the mRNA expression levels of PepT1 and ZnT1 in the small intestine. Meanwhile, the absorption mechanism of the complexes in the small intestine was also discussed.

## 2. Materials and methods

### 2.1. Materials

Soy meal (Shandong Yuwang Industrial Co. LTD, China), PP (KYKRQRW) was synthesized by Qiangyao Biological Technology Co., Ltd. Alcalase, papain, and neutral protease were obtained from Novozymes Biotechnology Co., LTD. Caco-2 cells (TCHu146) were purchased from Cells bank, Shanghai Chinese Academy of Sciences, CCK8 was obtained from MedChemExpress, Shanghai. All chemical reagents were analytically pure. Kunming male mice (KM) were obtained from the Animal Experiment Center of Guangdong University of Chinese Medicine. Zinc deficiency feed was purchased from Nantong Tuofei Feed Technology Co. LTD. Biochemical kits were purchased from Nanjing Jiancheng Biotechnology Co. LTD, China.

### 2.2. Preparation of SMHs

Soy meal solution (10%, w/v) was incubated in a 55°C water bath, and the pH was adjusted to 8. Alcalase, neutral protease, and papain (E/S = 1:50) were added, respectively. The hydrolysate was boiled for 15 min after equilibrating for 8 h. Finally, the cooling hydrolysate was centrifuged at 8800 g, and the supernatant was filtered by an ultrafiltration membrane (3KD MW) to obtain SMHs. The filtered liquid was dialyzed for 12 h with a 100 Da dialysis tube to remove salts. The residual liquid was finally freeze-dried for further use.

### 2.3. Preparation of SMHs-Zn complexes

The preparation of SMHs-Zn was based on previous studies with some modifications (18). SMHs powder was dissolved in distilled water at a concentration of 2 mg/mL, with the ZnSO_4_ added at different mass ratios (4:1–10:1). The mixture was then incubated at different temperatures (35–75°C) for 30–120 min under stirring. After equilibration, the mixture was centrifuged at 8,800 g for 15 min, and the supernatant was collected and dialyzed for 12 h using a 100 Da dialysis bag. The residual liquid was finally concentrated by rotary evaporation and freeze-dried.

### 2.4. Determination of Zn content

SMHs-Zn (0.5 g) powder was digested by H_2_NO_3_ (50%) solution using a digestion furnace (HYP-3, Shanghai). A colorimetric assay was applied to determine the Zn-binding capacity of peptides with 5-Br-PADAP as a color agent, referring to a previous study (19). The Zn content of SMHs-Zn (C_0_) and SMHs (C_1_) was measured at 556 nm. SMHs were used as the control.

The Zn (1 mg/ml) standard solution was used for the standard curve. It was proved that the results showed no difference with ICP-MS. The Zn-binding capacity was calculated as:


$$\mathrm{Zn}-\mathrm{binding capacity}\;\left(\%\right)=\frac{\left(C_{0}-C_{1}\right)}{\left(\mathrm{mass of the substrate}\right)}\times 100$$

### 2.5. Characterization of SMHs-Zn complexes

#### 2.5.1. UV spectrum analysis

The SMHs and SMHs-Zn powders were dissolved in deionized water at 0.1 mg/mL. A UV spectrophotometer (UV2600, Shimadzu Co., Ltd., Japan) was employed to determine the UV spectrum.

#### 2.5.2. FTIR assay

The spectra of different samples were recorded by FTIR instrument (Bruker Corporation, Germany). The SMHs and SMHs-Zn samples (1 mg) were blended with KBr powder and then pressed into a thin film and scanned in the range of 400–4,000 cm^−1^.

#### 2.5.3. Fluorescence spectroscopy

A fluorescence spectrophotometer (F-7000; Japan) was employed to investigate the secondary structure changes of the SMHs after binding with Zn ions. SMHs-Zn were prepared at different concentrations of Zn (10, 8, 6, 4, 2, and 0 mmol/L). Under the wavelength scanning model, SMHs-Zn samples were scanned at 280 nm of excitation wavelength and 300–480 nm of emission wavelength (20).

#### 2.5.4. Surface hydrophobicity

ANS (8-anilino-1-naphthalenesulfonic acid) fluorescence probe method was used for determination. The sample was diluted to different concentrations (0.2–1 mg/mL) with phosphate buffer solution (10 mM, pH 7.0). Then, 40 μL ANS solution (8.0 mM) was mixed with 3 mL diluted sample solution, and the fluorescence intensity was measured by fluorescence spectrometer (F-7000; Japan). The excitation wavelength was 360 nm, and the emission wavelength was 515 nm. Both the excitation and emission slits were 5.0 nm. The slope (S0)was used as an index of protein hydrophobicity.

#### 2.5.5. Morphology and element composition (SEM-EDS)

The morphology of SMHs and SMHs-Zn was observed by a JSM-7001F field emission scanning electron microscope (JEOL Co., Ltd., Japan). SMHs and SMHs-Zn powders were placed on the conductive tape which was fixed on a copper stage and then coated with gold and observed by SEM at a 15 kV accelerating potential.

#### 2.5.6. Solubility and dynamic light scattering of Zn at different pH values

The Zn solubility of the SMHs-Zn and ZnSO_4_ was measured by the previous method with some modifications (9). The SMHs-Zn and ZnSO_4_ samples were dissolved in citric acid phosphate buffers at pH 2.5–8.5 at 1 mg/mL, respectively. Incubated in a shaker at 25°C for 60 min, the Zn content of the supernatant was determined after centrifuging at 8000 g. And the supernatant was further analyzed with a Nano-ZS90 (Malvern Instruments Ltd., United Kingdom). The Zn solubility was calculated as:


$$\mathrm{Zn}\;\mathrm{solubility}\;\left(\%\right)=\frac{\mathrm{Zn}\;\mathrm{in supernatant}}{\mathrm{Initial}\;\mathrm{Zn}\;\mathrm{content}}\times 100$$

#### 2.5.7. CCK-8 assay

The Caco-2 cells is a human-cloned colonic adenocarcinoma cells that resembles differentiated small intestinal epithelial cells in structure and function. It was applied to investigate the toxicity of SMHs-Zn to the small intestine in this study. The CCK-8 assay was used to observe the proliferation of the cells according to previous publications with some modifications (10). Normal saline was used as a blank control.

### 2.6. Animal experiments

#### 2.6.1. Method of administration

All animal experiments followed the guidelines of EU Directive 2010/63/EU for animal experiments and were approved by the Guangzhou University of Traditional Chinese Medicine, China (Approval Number: SYXK (Guangdong) 2018–0034). Ninety healthy male Kunming mice 6 weeks of age (about 20–25 g) were randomly divided into nine groups. After a 2-week adaptation period, the blank group was treated with deionized water, and four groups underwent supplementation with SMHs-Zn-L (1.74 mg Zn/kg body weight), PP-Zn-L (1.74 mg Zn/kg body weight) and SMHs-Zn-H (6.96 mg Zn/kg body weight) and PP-Zn-H (6.96 mg Zn/kg body weight). The other four groups were administered with SMHs (consistent dose with SMHs-Zn-H), PP (consistent dose with PP-Zn-H), ZnSO_4_ (6.96 mg Zn/kg body weight), and zinc gluconate (6.96 mg Zn/kg body weight) as the controls. The dose of Zn was selected according to animal standards (GB14924.3–2010). All groups were fed with a zinc-deficiency diet (mice were given the AIN-93 recommendation diet, containing 1 ppm zinc) and deionized water freely, intragastric administration for 21 days. The body weight was recorded (3 d/cycle).

After the period of administration, the mice were killed by intraperitoneal injection of sodium pentobarbital solution, and the blood, liver, kidneys, pancreas, thymus, and testis were collected. Whole blood was placed at 25°C for 30 min before centrifugation (3,300 g), and the serum (50 μL) was collected and stored at −80°C with other organs for further study. Organs were weighted and treated with a tissue homogenizer, and the small intestine contents were dried and weighed for further use.

#### 2.6.2. Quantification of Zn in the serum, liver, and the small intestine contents

Zn levels in the serum, liver, and small intestine contents were measured by the zinc concentration detection kit (Shanghai Hengyuan Biotechnology Co., LTD) to observe the Zn absorption and metabolism in mice.

#### 2.6.3. Biochemical assays

All the Biochemical indicators were measured following the kit instructions (Shanghai Hengyuan Biotechnology Co., LTD). Including levels of superoxide dismutase (SOD) and malondialdehyde (MDA) of liver, SOD, alkaline phosphatase (AKP), and the total protein (TP) and albumin in serum.

#### 2.6.4. qPCR assay

The mRNA expression levels of PepT1 (Slc15a1) and ZnT1 (Slc30a1) are responsive to the peptide complexes and Zn under physiologically relevant conditions and reflect the absorption of peptide and Zn in the small intestinal epithelial cells (21, 22). In this study, qPCR was conducted with the SYBR Green method using a real-time fluorescence quantitative PCR instrument (CFX96, bio-rad, United States). The mRNA expression levels of PepT1 and ZnT1 were determined with β-actin as an endogenous control.

### 2.7. Statistical analysis

All experiments were conducted in triplicate and data obtained were analyzed by single factor ANOVA test with an SPSS 26.0 software (IBM), *p*<0.05. Origin 8.0 was used for the Figures. Bio-Rad CFX Manager was use for qPCR analysis.

## 3. Results and discussion

### 3.1. The formation of SMHs and SMHs-Zn complexes

The degree of hydrolysis (DH) plays an important role in the metal binding ability of the peptides obtained from food proteins (23). Based on the results of the previous study (20), in order to improve the hydrolysis of soy meal to obtain more Zn complexing sites, Alcalase, neutral protease, and papain were used. From Supplementary Figure S1, the hydrolysis degrees of soy meal was 29.04%, higher than in previous studies (24).

The amino acid composition of SMHs and PP was shown in Supplementary Tables S1,2. According to previous studies, amino acids can produce electrostatic interaction or coordination reactions with metal ions, including Asp, Ser, Glu, His, Lys, and Arg (6). We found that the total value of these amino acids reached 49.81% in soybean peptides, which to some extent determined the binding ability of the peptide zinc. The metal-binding amino acid in PP reached 53.73%, and the Zn-binding capacity in PP-Zn was 83.21%, higher than that of SMHs-Zn, which may be related to various factors. The factors affecting the metal binding ability was not only the amino acid composition and content of peptide but also the metal-binding amino acid sequence and position, their varying binding strengths, configuration, and other factors (25). For example, the same number of metal-binding amino acids in Glu-Pro-Ser-His (Glu, Ser, and His) as that in Asn-Ser-Met (Asn, Met, and Ser), they did not exhibit the same Zn-binding capacity (26). One explanation for this might be that Asn and Met are stronger chelators than Glu and His, and another explanation could be the position of Asn in the peptide Asn-Ser-Met (26).

During the complexation process, it was observed that the temperature, pH, reaction time, and mass ratios of SMHs and Zn had great influences on the Zn-binding rate of SMHs-Zn, Supplementary Figures S2A–D. While SMHs and ZnSO_4_ were mixed at 65°C, pH 5.0–6.0, 90 min, and a mass ratio of 4:1, resulting in a higher binding capacity (12.45 ± 0.46%).

### 3.2. The binding mechanism of SMHs-Zn complexes

#### 3.2.1. UV spectrum

**Figure 1:**
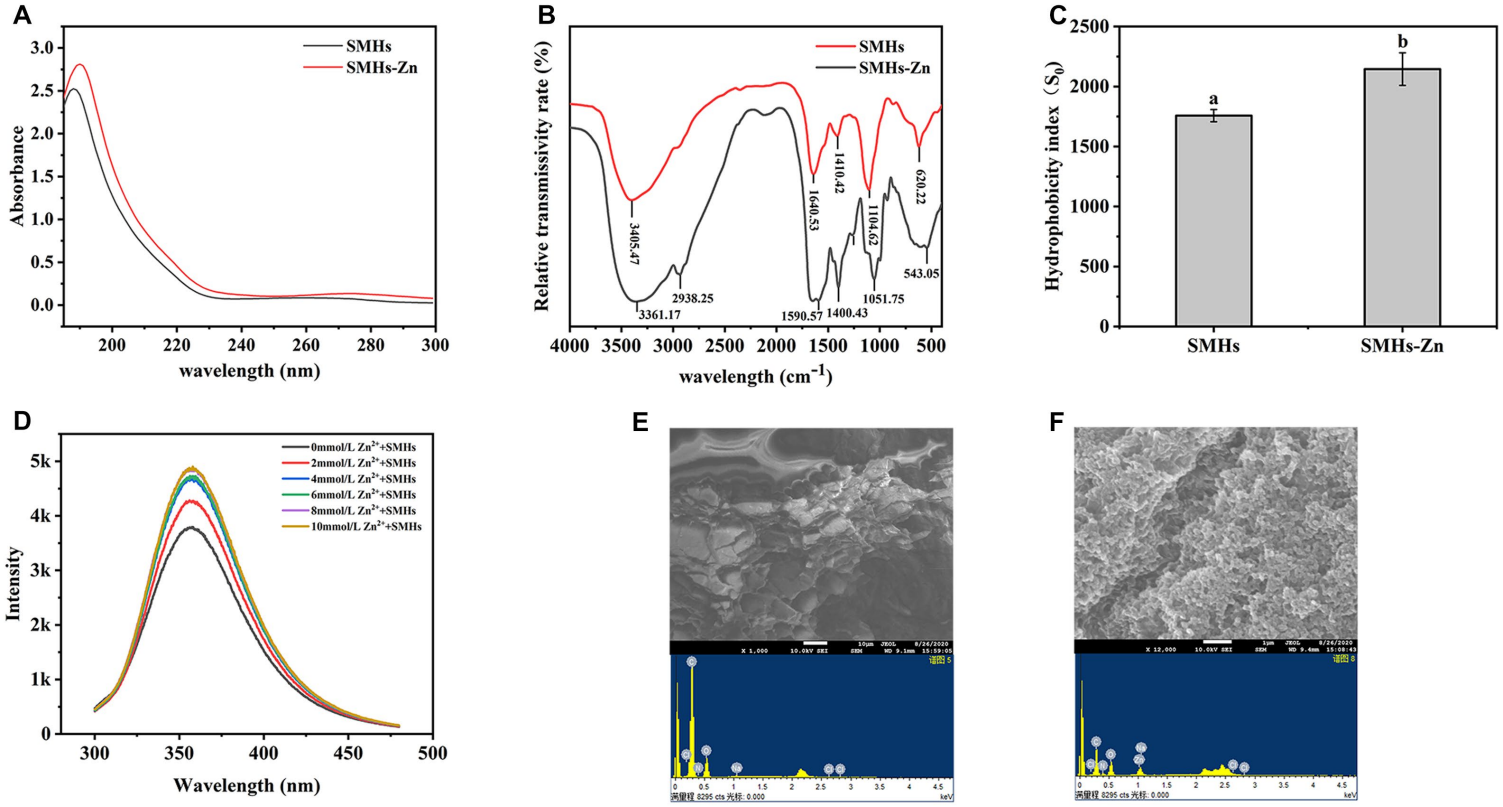
UV spectrum of the SMHs and SMHs-Zn **(A)**, FTIR spectra of the SMHs and SMHs-Zn **(B)**, and the hydrophobicity of SMHs and SMHs-Zn **(C)**, Fluorescence spectrum of the SMHs-Zn **(D)**, SEM-EDS observation of the SMHs **(E)** and SMHs-Zn **(F)**.

UV spectroscopy assay was employed to investigate the formation mode of SMHs-Zn complexes. As shown in Figure 1A, the maximum absorption peaks at 187 nm were red-shifted to 191 nm after binding with Zn. The binding of organic ligands and metal ions induced the shifting or enhancement of absorbance peaks, which might be caused by the relevant valence electron transition (8). When the absorption peak of the protein was enhanced, it could be considered that Zn entered the hydrophobic cavity of the peptide, leading to the extension of the peptide chain with the exposure of the chromogenic groups and the improvement of hydrophobicity (Figure 1C).

#### 3.2.2. FTIR assay

Infrared spectroscopy is a mature characterization method to explore the interaction between peptides and metal ions. As shown in Figure 1B, SMHs exhibited typical amide band peaks; the peak at 1640.525 cm^−1^ was caused by C=O, C-N stretching (Amide I and II), blue-shifted to 1590.569 cm^−1^ indicative of C=O, C-N involved in the binding of Zn (11). The peak that appeared at 3405.468 cm^−1^ was caused by -NH and -COOH on the side chain; it was blue-shifted to 3361.167 cm^−1^ because -N could form coordination bonds with Zn ions by providing electron pairs, hydrogen bonds were replaced by Zn-N bonds, and -COOH possessed a negative charge that was likely to bind with cationic Zn (23). The absorption peaks at 620.22 cm^−1^ was the amine IV band, and the red-shift to 543.05 cm^−1^ was caused by the increase in the electron cloud density of the O=C-NH bond and the decrease in the electron cloud density of the -NH bond (27). All this evidence indicated that Zn was bound with carboxyl oxygen and amino nitrogen atoms on SMHs, bridged by Zn-O and Zn-N bonds.

#### 3.2.3. Analysis of endogenous fluorescence spectra

By analyzing the position and intensity changes of the absorption peaks of these groups, we can understand the changes in the spatial structure of peptides (28). As shown in Figure 1D, with the increase in Zn content, the fluorescence intensity of SMHs-Zn increased gradually due to the changes in the microenvironment of fluorescent groups. It was worth noting that the fluorescence increasing was the most significant at 0–2 mmol/L and reached saturation at 10 mmol/L, consistent with the results in Supplementary Figure S2. The chelation rate was highest when the mass ratio of SMHs to Zn was 4:1. A previous study found that amino acids such as Asp., Ser, Glu, His, Lys, and Arg had strong metal-binding capacity (6). When Zn was added to peptides, the binding effect changed the structure of the peptides and exposed the fluorophores (Tyr and Phe), leading to an increase in fluorescence intensity. Based on this evidence, it was speculated that the Zn-binding effect induced the folding of the SMHs and changed the fluorescence energy transfer (29).

#### 3.2.4. SEM-EDS assays

SEM-EDS was used to investigate the morphology and elemental composition of SMHs and SMHs-Zn. As shown in Figure 1E, SMHs exhibited a flake and honeycomb structure, while SMHs-Zn showed a tight and grainy structure with a nanoscale in Figure 1F, indicating that the formation of SMHs-Zn affected the structure of native hydrolysate. In Figures 1E,F, it was observed that SMHs were mainly composed of C and O (13.22 and 86.78%) with 8.70% Zn detected in SMHs-Zn, which indicated that Zn was bound with SMHs.

#### 3.2.5. Zn solubility of SMHs-Zn at different pH values and DLS assay

**Figure 2:**
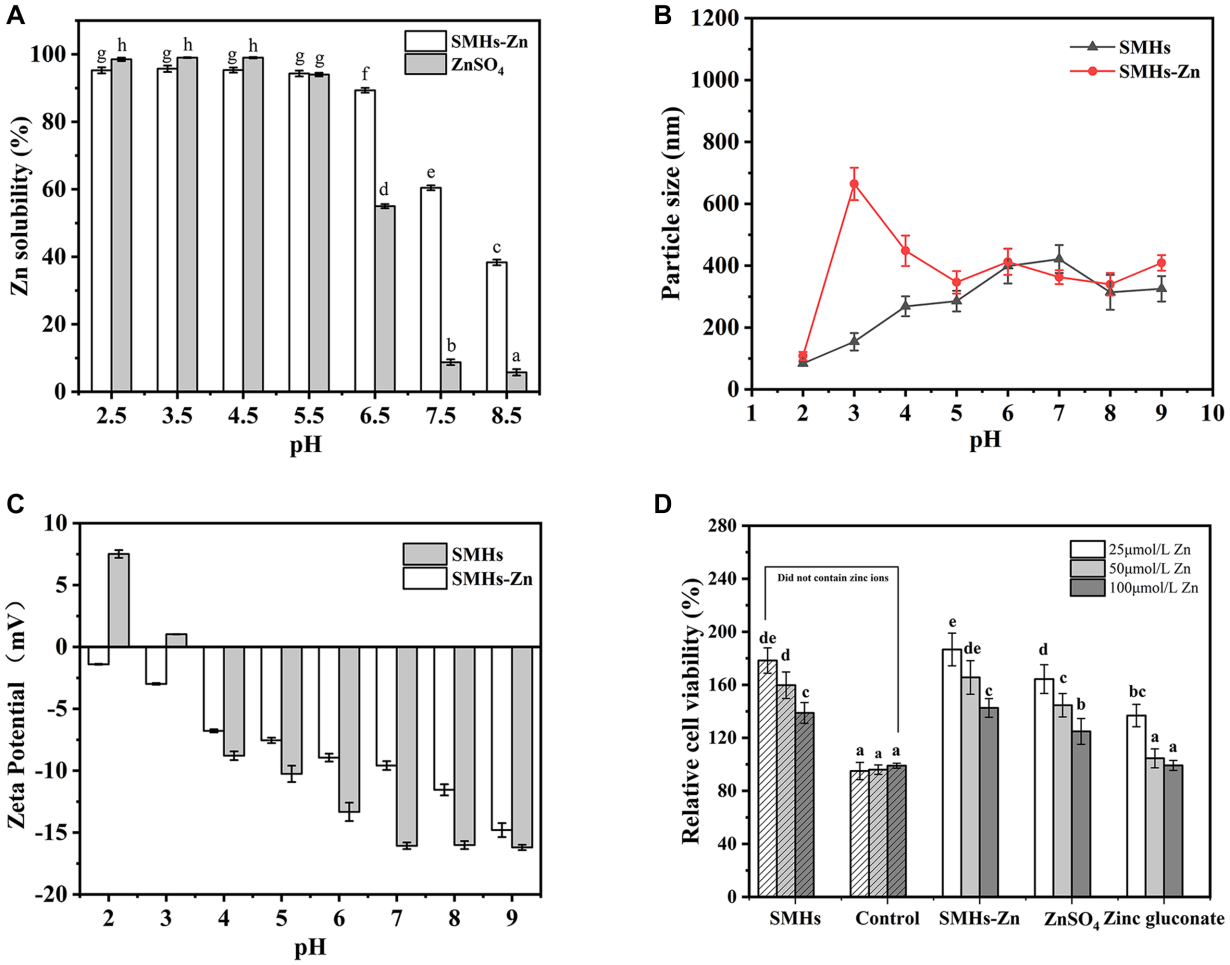
Zn solubility at pH 2.5–8.5 **(A)** of the SMHs-Zn and Particle size **(B)** and Zeta Potential **(C)** of the SMHs-Zn at pH 2.0–9.0 and Caco-2 cells viability test of the SMHs-Zn **(D)**.

Zn solubility is an important character to evaluate the absorption efficiency of Zn in the human body. As shown in Figure 2A, all of the samples exhibited high Zn solubility at pH 2.5–5.5, but reduced gradually at pH 6.5–8.5 because of the formation of Zn(OH)_2_. Although the solubility of SMHs-Zn significantly decreased at pH 7.5, the zinc-releasing percentage was 60.45 ± 0.71%, but it was still significantly higher than ZnSO_4_ (8.76 ± 0.85%). This is similar to the pH of the human gastrointestinal environment. SMHs-Zn possessed higher Zn solubility (*p* < 0.05) than ZnSO_4_ at alkaline conditions, which indicated that Zn was protected by SMHs and improved the bioavailability of zinc in the human gastrointestinal tract.

DLS was used to characterize the particle sizes and surface charges of SMHs-Zn at pH 2.0–9.0. As shown in Figure 2B, all samples showed a small particle size (<650 nm) at pH 2.0–9.0 after centrifuging. It revealed that SMHs-Zn had small particle sizes under various pH conditions which might be beneficial for absorption by cells (18). In Figure 2C, SMHs showed negative charges at pH 4.0–9.0 which might be susceptible to electrostatic interaction with Zn. It was observed that the complexation improved the charges of SMHs, because Zn^2+^ neutralized some negative charges (18).

#### 3.2.6. Cytotoxicity test *in vitro*

As shown in Figure 2D, all of the samples promoted cell proliferation at low concentrations, which indicated that both SMHs and Zn were beneficial for cells growth. With the increase in Zn content in the samples, the proliferation effect of cells decreased significantly, but presented no toxicity to the cells, only a high concentration of Zinc gluconate inhibited the cells growth. Moreover, compared with organic and inorganic Zn, SMHs, and Zn complexes were suggested to possess better biological activity on cells growth, achieving 180%, with a certain coordination promotion effect. Therefore, it was suggested that food-derived hydrolysate and the Zn complexes possessed high biological activity beneficial to Caco-2 cells (8), for the reason that ZnSO_4_ was easy to precipitate, and a high content of Zinc gluconate was toxic to cells.

### 3.3. The effect of SMHs-Zn and PP-Zn complexes on zinc absorption in mice

#### 3.3.1. Effects of Zn-complexes on the growth of mice

**Figure 3:**
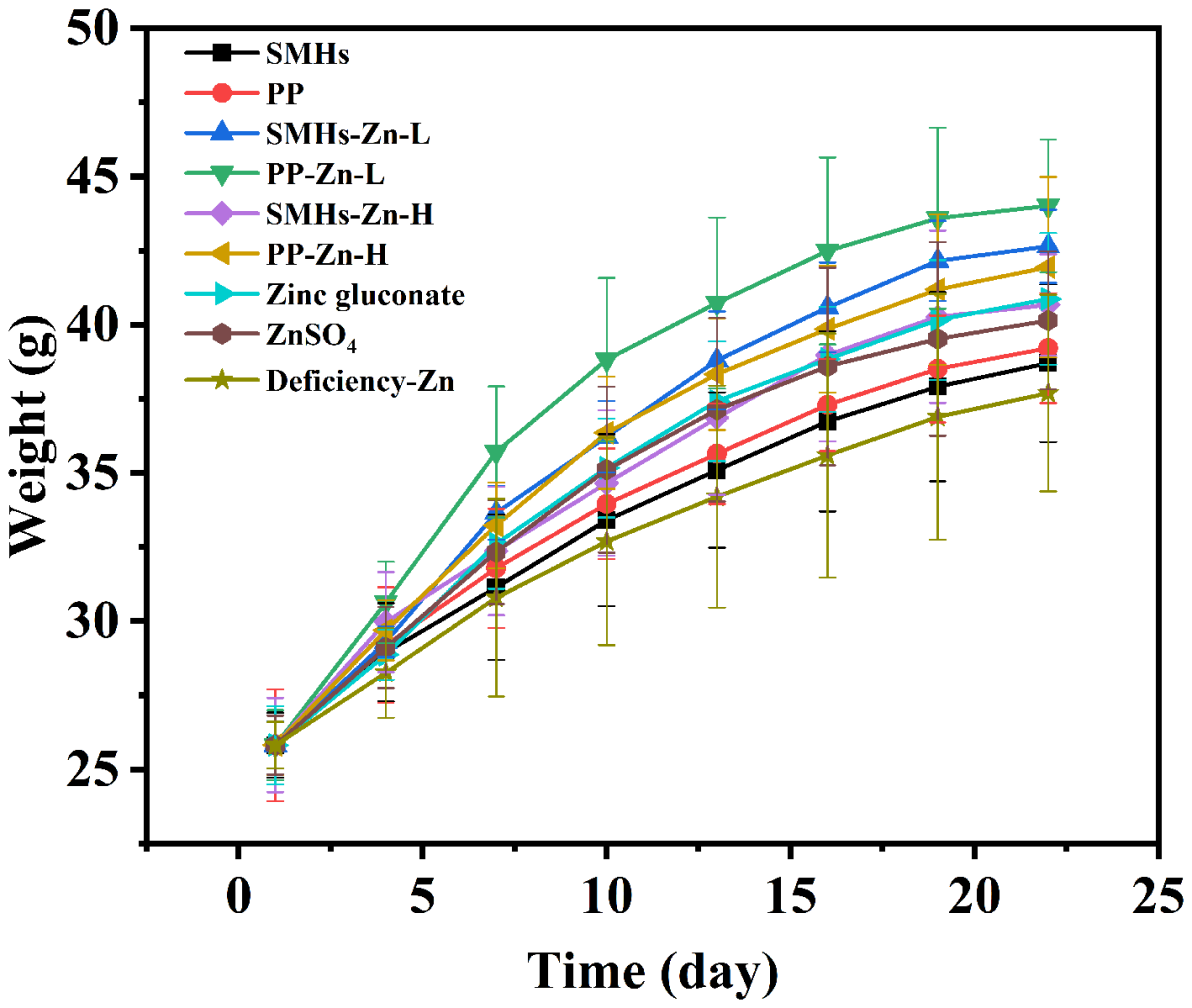
Growth curves of mice fed with different Zn supplements.

**Figure 4:**
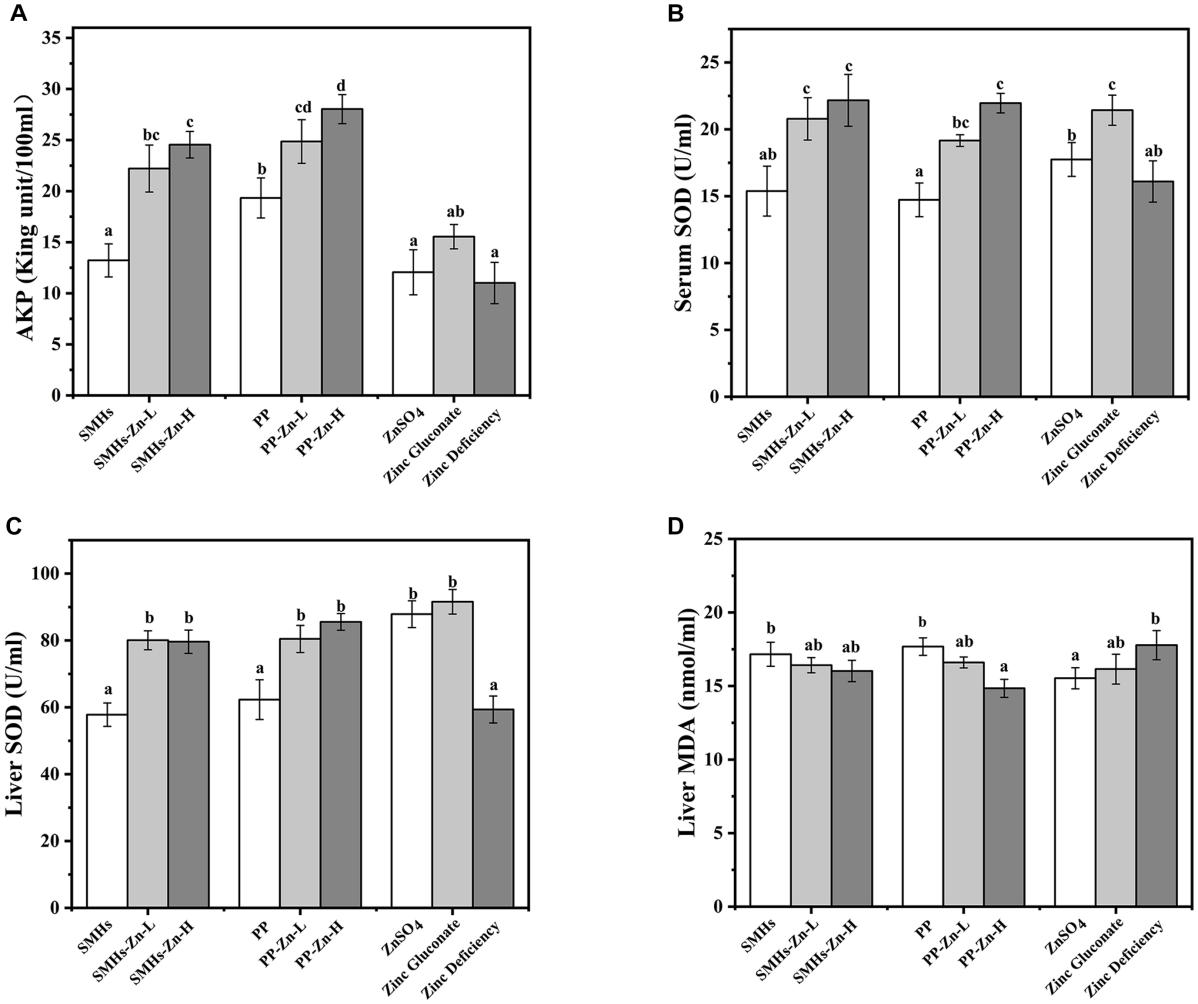
AKP **(A)** and SOD **(B)** activity of the serum, SOD **(C)** activity and MDA **(D)** content of the liver.

The supplemental form of Zn plays an important role in its absorption. The same amount of Zn can have different absorption effects in organism (30). As shown in Figure 3, Zinc deficient mice showed growth retardation; supplementation of different kinds of zinc could improve the growth of mice; and the promotion effects were as follows: PP-Zn-L>SMHs-Zn-L>SMHs-Zn-H, PP-Zn-H, Zinc gluconate>ZnSO_4_>PP>SMHs>Zinc deficiency. Body weight in PP-Zn-L and SMHs- Zn-L was higher than that of PP-Zn-H and SMHs-Zn-H in mice, which also indicated that the absorption efficiency of the peptide zinc complex is high, and the low dose of zinc intake can meet the normal growth needs of mice. However, PP-Zn-H and SMHs- Zn-H groups did not reduce the activity of AKP and SOD on related enzymes in the serum and organs, and the content of MDA was controlled at a low level (Figures 4A–D). This indicated that PP-Zn-H and SMHs-Zn-H did not cause damage to the enzyme system in mice. Previous studies found that both Zn deficiency and high Zn can alter intestinal and absorptive decrease in the feed intake (31, 32). Dietary Zn is an essential mediator of microbial community structure, and both zinc deficiency and excess can cause an imbalance in gut microbial levels (33, 34). This may be one reason why the mice decrease in the body weight. The results showed that PP-Zn and SMHs-Zn could be better absorbed and utilized by mice, and they were better utilized by mice.

#### 3.3.2. Zn supplementation of SMHs-Zn and PP-Zn complexes In mice

**Figure 5:**
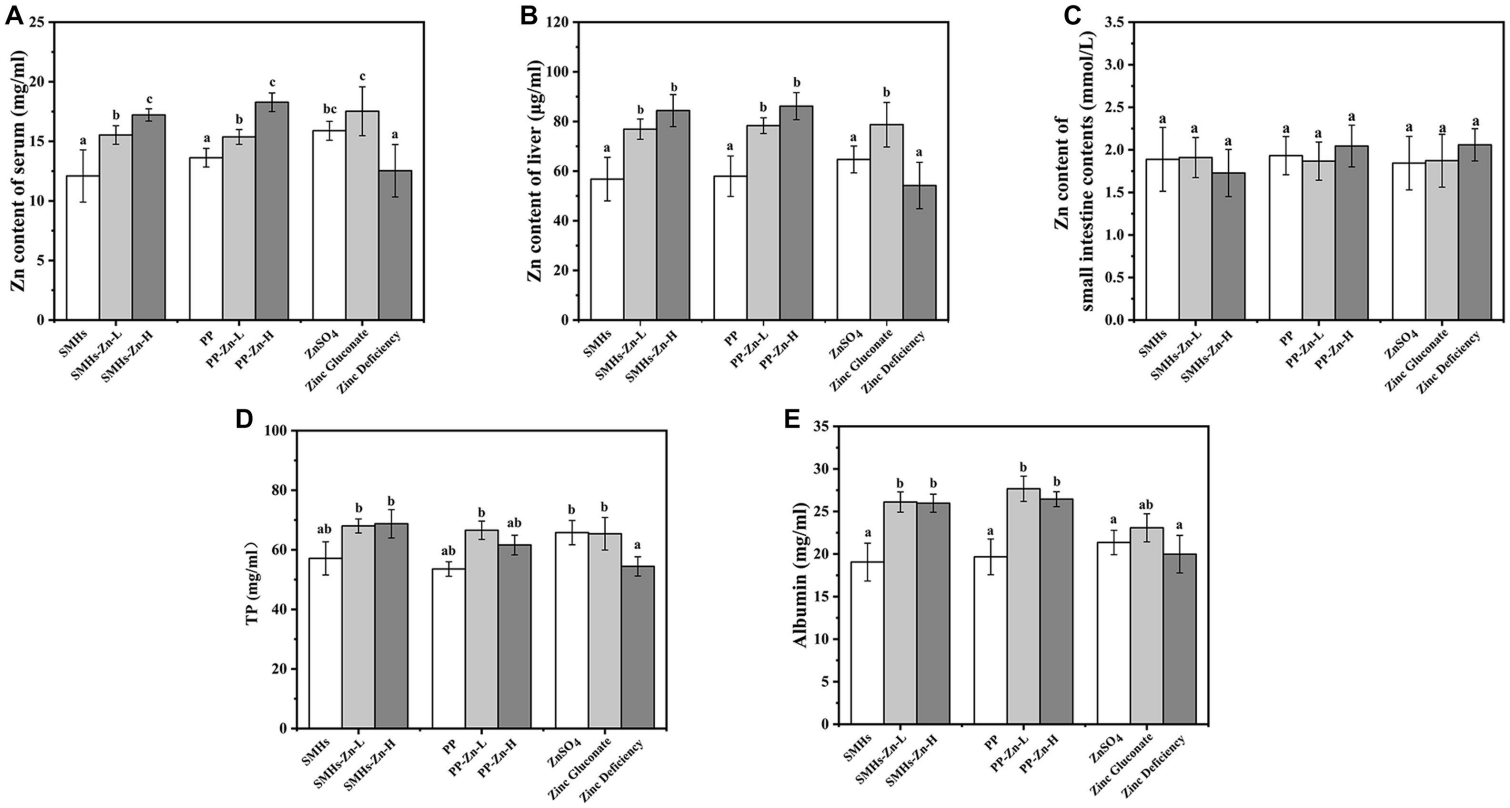
Zn content of the serum **(A)**, liver **(B)** and the small intestine contents **(C)**, and total protein **(D)** and albumin content of serum **(E)**.

As shown in Figures 5A,B, the accumulation of Zn in the liver and serum of mice fed the Zn complexes increased significantly with dose dependence, compared with the controls. It revealed that feeding mice with peptide-Zn complexes was more effective in absorption, which was similar to the effect of organic Zn but better than that of inorganic Zn. And there was no significant difference in the free Zn content in small intestine content (Figure 5C), suggesting that peptide-Zn were effectively absorbed. Moreover, mice fed with Zn supplements showed significant high levels of TP and albumin; SMHs-Zn, PP-Zn, and Zinc-gluconate displayed higher contents (Figures 5D,E). The increase in TP and albumin contributed to the absorption and transportation of peptide-Zn complexes, which indicated that peptide-Zn possessed higher bioavailability in the small intestine and needed more proteins for Zn absorption and transportation (1). In general, peptide-Zn complexes had a high solubility and nanoscale size in the small intestine resulting in higher availability than inorganic Zn, as they could be absorbed directly.

#### 3.3.3. Effects of SMHs-Zn and PP-Zn complexes On related enzymes In The serum and organs

According to Figure 4A, it was observed that the activity of AKP in the serum of the SMHs-Zn and PP-Zn groups were significantly enhanced, compared with the controls. It was reported that AKP was regarded as a Zn (II) metalloenzymes, whose activity was affected by dietary Zn (35). The increase in AKP was beneficial to the growth of mice. Moreover, the SOD activity in the serum and liver (Figures 4B,C) increased significantly, similar to previous studies (36). However, there was no significant dose-dependent enhancement of SOD activity in mice with SMHs-Zn and PP-Zn groups, and there was no significant difference between the effect of the SMHs-Zn and PP-Zn, but the effect was stronger than that of ZnSO_4_. As for the MDA levels of the liver in Figure 4D, they were similar across all groups, indicating that no oxidative damage occurred in the mice. Yuan et al. found that shrimp fed the zinc amino complexes showed higher antioxidant ability and immunity, as a result of the increased activity of related enzymes *in vivo* (37). These results revealed that SMHs-Zn and PP-Zn could be more effectively absorbed by the small intestine than organic and inorganic Zn. And the absorbed Zn was further utilized by organs for AKP, SOD, and synthesis or activation, which consisted with the higher serum zinc content in serum and liver. But there were no statistical differences found between the SMHs-Zn and PP-Zn diets, and peptide-Zn complexes did not show a significant dose-dependence effect on enzyme activity.

#### 3.3.4. qPCR assay of PepT1 and ZnT1 on mRNA expression levels

**Figure 6:**
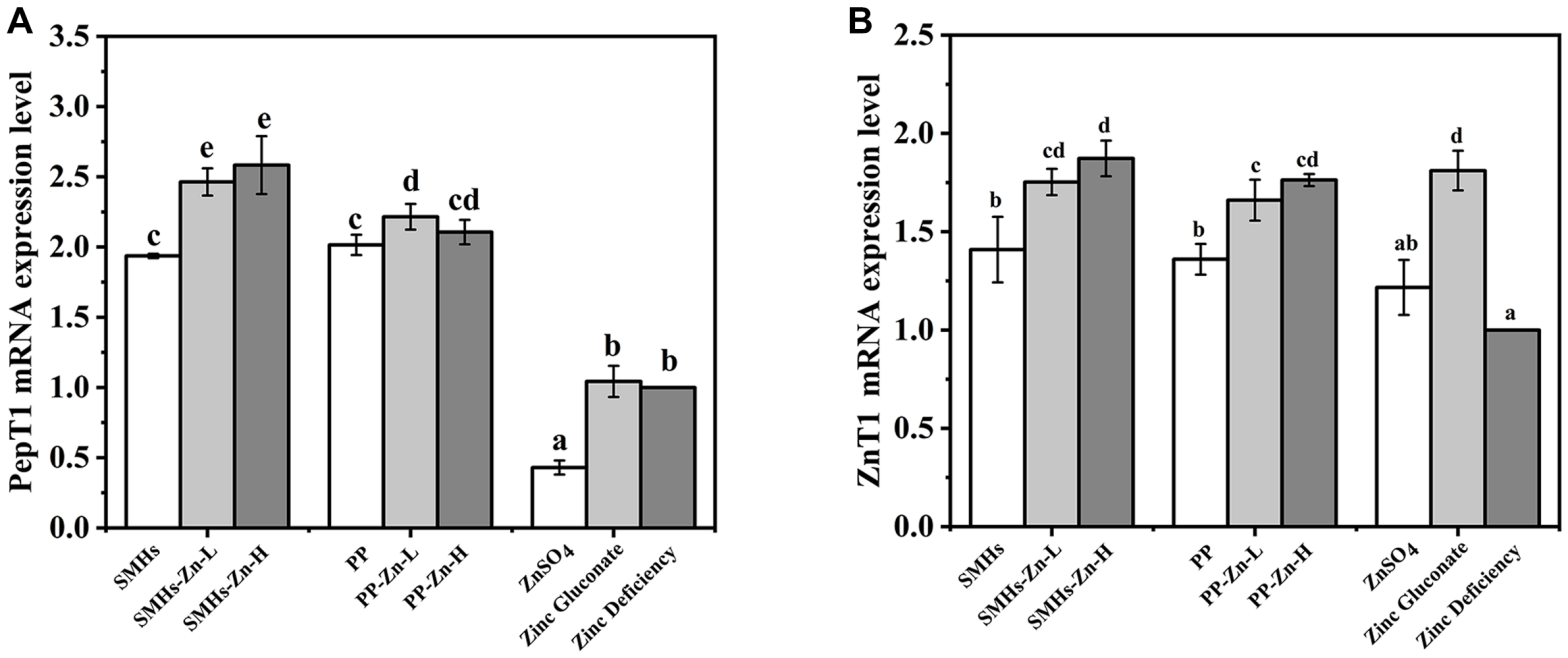
The mRNA expression levels of PepT1 **(A)** and ZnT1 **(B)** in the small intestine.

The PepT1, located at the apical side of small intestinal epithelial cells, is a transporter that mediates the transport of peptide complexes. It is the most thoroughly studied and widely used transporters among peptide transporters at present (38). Figure 6A showed the qPCR results of different Zn supplements on the mRNA expression levels of PepT1. Mice fed with SMHs and PP exhibited higher PepT1 mRNA expression levels than ZnSO_4_ and Zinc gluconate. It seemed that the peptide source stimulated the expression of PepT1, whereas there was no significant difference between PP and SMHs. The SMHs-Zn and PP-Zn complexes might be absorbed by PepT1 as a whole. It was an active transport process that was different from the absorption of ZnSO_4_, and it increased the digestibility and availability of the complexes. Furthermore, SMHs-Zn possessed notably higher PepT1 mRNA expression levels than the original peptides. It was reported that PepT1 has a better affinity for hydrophobic peptides but is almost unable to bind to dipeptides with two positive charges (39). Furthermore, selected amino acids (leucine and phenylalanine) were shown to control rat PepT1 promoter activity via the amino-acid-responsive element (AARE) localized upstream of the start codon (40). SMHs-Zn had a more abundant amino acid composition after intestinal digestion, which resulted in better PepT1 mRNA expression levels than PP-Zn (41).

It has been shown that the peptide-Zn complexes are absorbed by transporters such as PepT1 through active transport and then released into the blood by ZnT1 (42). To ensure sufficient capacity for the export of Zn into the portal blood and control intracellular Zn levels, the mRNA expression levels of ZnT1 was Zn-dependent (43). It was observed that the ZnT1 mRNA expression levels of the SMHs-Zn, PP-Zn, and Zinc gluconate groups were significantly higher than ZnSO_4_ and the controls, in Figure 6B. The results directly reflected that the zinc absorption of the complexes was significantly higher than that of zinc sulphate. It was speculated that the inorganic Zn was not stable in the small intestine, which affected its absorption. Conversely, SMHs-Zn and PP-Zn complexes were absorbed effectively, as well as organic Zn through the PePT1 receptor channel, and further released into the blood by ZnT1. In addition, SMHs-Zn with lower Zn-binding capacity had slightly higher mRNA expression levels than PP-Zn. It might be affected by the composition of amino acids; SMHs-Zn complexes contained many types of amino acids, which were utilized as nutrients preferentially by the intestine. In previous studies (44–46), it has been found that zinc absorption in the small intestine may be affected by a variety of antagonists, such as phytic acid, iron ions, calcium ions, polyphenols, etc. Peptide-Zn complexes with different amino acids were affected differently by antagonists. Glu-Zn was less affected by absorption inhibitors than Lys-Zn and Met-Zn (47). This may be one of the reasons why the SMHs have a lower Zn-binding capacity than PP-Zn and there is no significant difference in the expression levels of ZnT1 mRNA between them.

## 4. Conclusion

In our results, Zn was mainly combined with -NH, -COOH, -OH groups of SMHs, with hydrophobic and electrostatic interactions as auxiliary stabilizing forces. SMHs-Zn was proved to have great solubility and small particle size at different pH values and it showed a benefited effect on Caco-2 cells growth. In the animal model experiments, the effect of SMHs-Zn complexes on zinc absorption in mice did not depend on its coordination ability, and the same dosage of hydrolysates-Zn complexes with lower Zn-binding capacity could achieve the same effect of PP-Zn complexes. PP-Zn had better effect on the growth of mice, but the mRNA expression levels of PepT1 and ZnT1 were lower than SMHs-Zn, indicating that it was related to the amino acid composition and properties of the hydrolysate. Furthermore, we established a transport and absorption model of peptide-zinc complexes based on PepT1 and ZnT1 transporters. Firstly, the peptide-Zn complexes were absorbed by the small intestinal epithelial brush cells through PepT1, and then transported to plasma by ZnT1 transporters on the base side, where it binds to albumin in plasma and was transported throughout the body in the form of conjugates for enzyme synthesis. The study can provide information on better understanding the bioavailability and absorption pathways of peptide-Zn complexes and hydrolysate-Zn *in vivo*.

## Data availability statement

The raw data supporting the conclusions of this article will be made available by the authors, without undue reservation.

## Ethics statement

The animal study was reviewed and approved by Guangzhou University of Traditional Chinese Medicine.

## Author contributions

RW and MY: investigation, data curation, software, and writing— original draft. SZ: supervision. QZ and YY: conceptualization, methodology, supervision, and writing—review and editing. All authors contributed to the article and approved the submitted version.

## Funding

This work was supported by Guangdong Provincial key R&D Program (No. 2022B0202030001) and Science and Technology Program of Guangzhou (No. 202201010700) and Open Project Program of China-Canada Joint Lab of Food Nutrition and Health, Beijing Technology and Business University (BTBU) (No. KFKT-ZJ-2107) and Guangzhou Science and Technology Program (No. 201903010108).

## Conflict of interest

The authors declare that the research was conducted in the absence of any commercial or financial relationships that could be construed as a potential conflict of interest.

## Publisher’s note

All claims expressed in this article are solely those of the authors and do not necessarily represent those of their affiliated organizations, or those of the publisher, the editors and the reviewers. Any product that may be evaluated in this article, or claim that may be made by its manufacturer, is not guaranteed or endorsed by the publisher.

## Abbreviations

SMHs: Soy meal hydrolysates
Zn: Zinc
PP: synthetic soy peptide (Lys-Tyr-Lys-Arg-Gln-Arg-Trap)
FTIR: Fourier transform infrared spectroscopy
UV: Ultraviolet–visible
DLS: Dynamic light scattering
SEM-EDS: Scanning Electron Microscope and Energy Dispersion Spectrum
AKP: Alkaline phosphatase
SOD: Superoxide dismutase
TP: Total protein
MDA: Malondialdehyde
qPCR: Quantitative Real-time PCR
