# Supplementary material for: Development, characterization and in vivo zinc absorption capacity of a novel soy meal hydrolysate-zinc complexes
Source: Front Nutr. 2023 Jul 6;10:1211609. doi: 10.3389/fnut.2023.1211609 (PMC10358849; doi:10.3389/fnut.2023.1211609)
Supplement: Supplementary file 1 [file Data_Sheet_1.docx]

Supplementary Material

Development, characterization and in vivo zinc absorption capacity of a novel soy meal hydrolysate-zinc complexes

Rongxin Wang Meijun Ye, Suyin Zhu, Qingzhu Zenga, Yang Yuan

*** Correspondence:** gzu228yuan@163.com

# Supplementary Figures and Tables

## Supplementary Figures


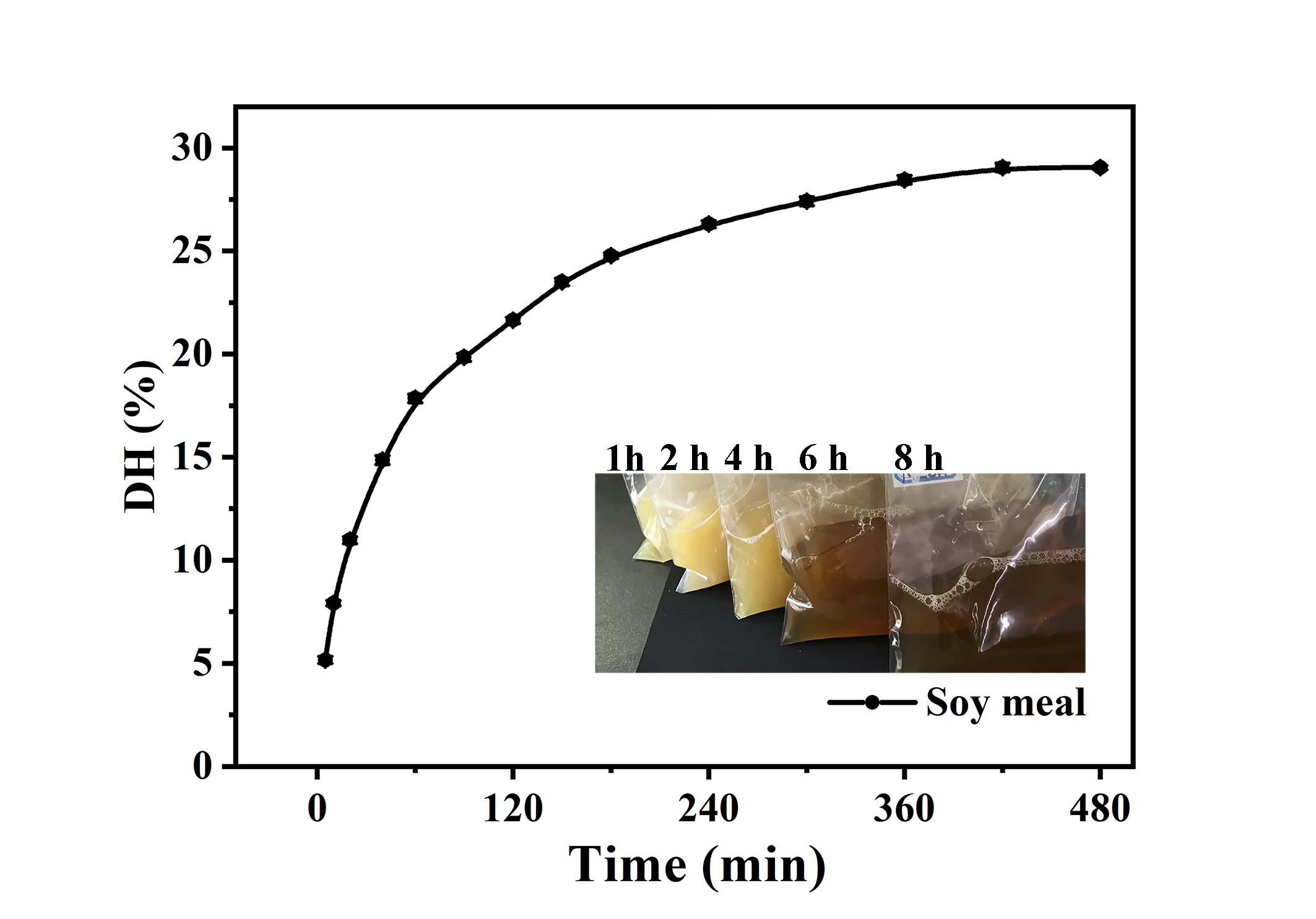


**Supplementary Figure 1.** The degree of hydrolysis of soy meal.

**
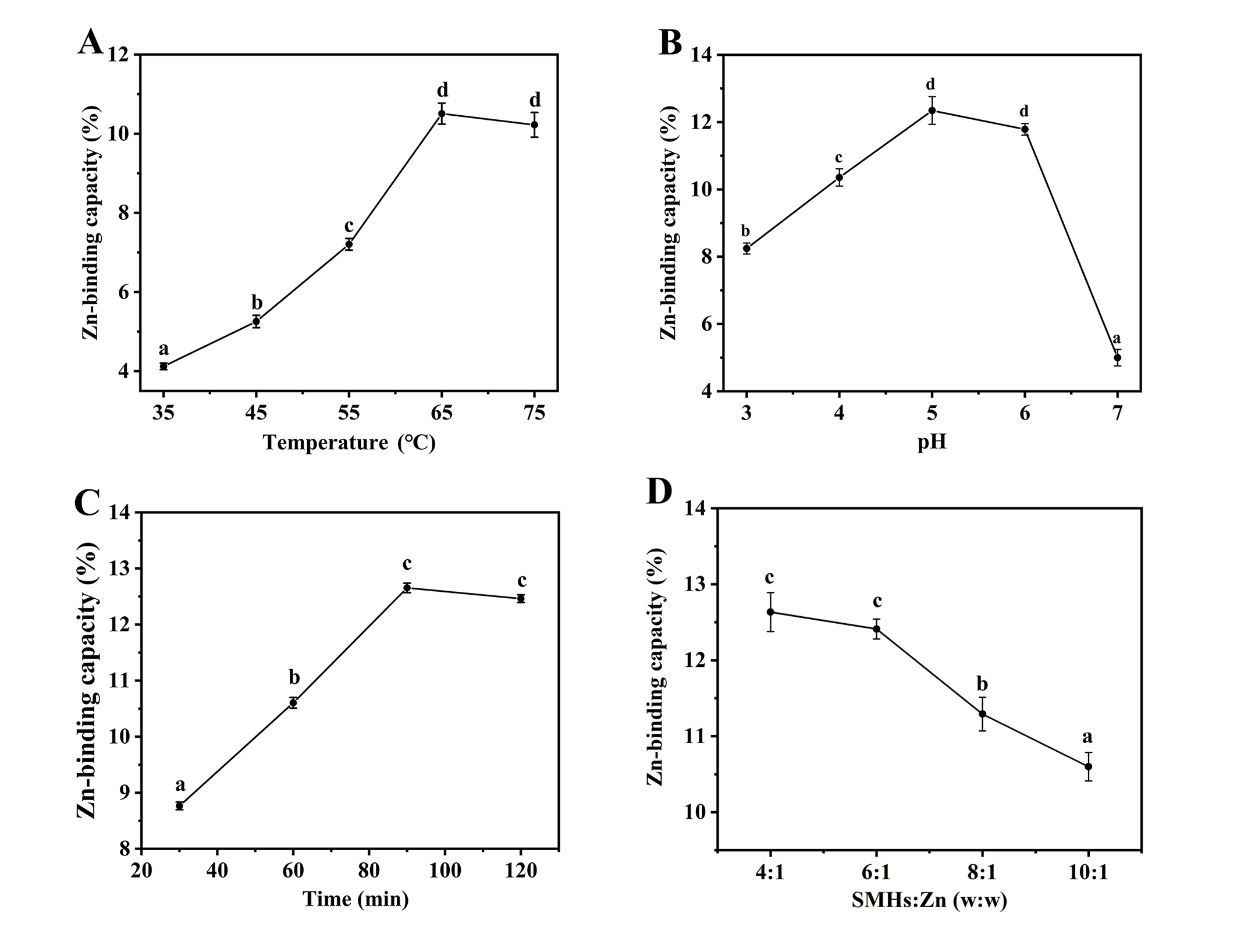
**

**Supplementary Figure 2.** Effects of temperature (A), pH (B), time (C), and SMHs: Zn mass ratios (D) on the Zn-binding capacity of SMHs-Zn.

## Supplementary Tables

**Supplementary Table 1.** Amino acid composition of SMHs.

| Sample | SMHs (%) |
| --- | --- |
| Asp | 24.51 |
| Ser | 10.6 |
| Glu | 16.78 |
| Gly | 2.06 |
| His | 5.67 |
| Arg | 21.87 |
| Thr | 16.98 |
| Ala | 14.62 |
| Pro | 3.63 |
| Cys | 4.18 |
| Tyr | 2.9 |
| Val | 7.06 |
| Met | 2.38 |
| Lys | 24.44 |
| Ile | 18.97 |
| Leu | 6.84 |
| Phe | 26.24 |
| Acidic amino acid % ^a^ | 19.80 |
| Basic amino acid % ^b^ | 22.93 |
| Aromatic amino acid % ^c^ | 13.71 |
| Essential amino-acid % ^d^ | 47.07 |
| Metal-binding amino acid % ^e^ | 49.81 |

^a^ including Asp and Glu; ^b^ including Lys, Arg, and His; ^c^ including Phe, Try, Tyr; ^d^ including Lys, Tyr, Phe, Met, Thr, Ile, Leu, and Cys; ^e^ including Asp, Ser, Glu, His, Lys and Arg

**Supplementary Table 2.** Amino acid composition of PP.

| Sample | PP (%) |
| --- | --- |
| Lys | 24.23 |
| Tyr | 15.31 |
| Arg | 29.50 |
| Gln | 12.02 |
| Trp | 17.47 |
| Acidic amino acid % ^a^ | 54.73 |
| Basic amino acid % ^b^ | 0 |
| Aromatic amino acid % ^c^ | 15.31 |
| Essential amino-acid % ^d^ | 39.54 |
| Metal-binding amino acid % ^e^ | 53.73 |

^a^ including Asp and Glu; ^b^ including Lys, Arg, and His; ^c^ including Phe, Try, Tyr; ^d^ including Lys, Tyr, Phe, Met, Thr, Ile, Leu, and Cys; ^e^ including Asp, Ser, Glu, His, Lys and Arg
